# Expression and Localization of Glycosaminoglycans/Proteoglycan in Pterygium: An Immunohistochemical Study

**Published:** 2019

**Authors:** Constantinos D. GEORGAKOPOULOS, Olga E. MAKRI, Dionisios PAGOULATOS, Nikolaos K. KARAMANOS

**Affiliations:** 1 Department of Ophthalmology, University of Patras, Medical School, Greece; 2 Biochemistry Laboratory, Department of Chemistry, University of Patras, Greece

**Keywords:** Glycosaminoglycans, Keratan Sulfate, Heparan Sulfate, Dermatan Sulfate, Pterygium, Immunohistochemical Study

## Abstract

Pterygium is a triangle-shaped fibrovascular hyperplasia of the bulbar conjunctiva on the cornea. The purpose of this study was to analyze Proteoglycans (PGs) by Immunohistochemistry (IHC) in pterygium tissues and to compare the results with normal conjunctiva. Twenty-four patients (14 males) undergoing primary pterygium excision and 17 healthy individuals (10 males), undergoing extracapsular cataract surgery, were included. Pterygium tissues and normal conjunctiva tissues were surgically removed. The tissue sections were fixed in 2% paraformaldehyde and incubated with monoclonal antibodies against PGs anti-mouse IgG. Immunohistochemical study showed stronger expression of keratan sulfate in the stroma of the pterygium compared to normal conjunctiva. An increased expression of heparan sulfate was observed in the epithelial layer and around the pterygium vessels. On the other hand, dermatan sulfate showed an increased expression and localization not only in the sub-epithelial area of the pterygium and normal conjunctiva, yet throughout the stroma of the pterygium. The differences in the expression and localization of the studied extracellular matrix proteoglycans in the pterygium tissue compared to normal conjunctiva may explain the tissue hyperplasia, structure, and the functional properties in pterygium.

## INTRODUCTION

Pterygium is a triangle-shaped fibrovascular hyperplasia of the bulbar conjunctiva on the cornea. It is one of the most common external eye diseases, especially in tropical countries and in people, who spend a significant amount of time outdoors [1]. Although the pathogenetic mechanism of pterygium is unclear, there are several hypotheses. Prolonged exposure to solar ultraviolet light is believed to be the main predisposing factor in the development of pterygium and the fact that it is usually detected nasally has been associated with light focused on this area [1, 2]. Several authors suggest that ultraviolet-induced alteration of limbal stem cells is an important factor in the invasive nature of pterygium. It is assumed that ultraviolet light promotes the transformation and overexpression of stem cells in the limbus [1-3]. It is suggested that mast cells are involved in the pathogenesis and progression of pterygium [4] while an immunopathogenetic mechanism, possibly type I and III hypersensitivity [5], and the overexpression of the Extracellular Matrix (ECM) molecular network have also been implicated [6]. Proteoglycans (PGs) comprise one of the most important constituents of the ECM with important regulatory roles in several cellular events and pathophysiological processes [7]. They contain one or more Glycosaminoglycan (GAG) chains that are covalently attached to a protein core and modify the functions of proteoglycans [8]. Glycosaminoglycans are linear heteropolysaccharides composed of repeated disaccharide units [7]. 

Although it is known that the tissue of pterygium contains PGs, a thorough analysis of the important connective tissue components has not been conducted so far. The purpose of the current study was to evaluate the expression and tissue localization in three different GAGs, i.e. keratan sulfate, heparan sulfate, and dermatan sulfate, in the pterygium and normal conjunctiva.

## METHODS

The study was conducted according to the tenets of the Declaration of Helsinki and received approval by the Institutional Review Board of the University Hospital of Patras. Exclusion criteria were any ophthalmic or systemic disease or use of topical or systemic medications. Informed consent was obtained from all eligible participants that participated in the study. 

Pterygium tissues were surgically removed, while normal conjunctiva tissues were obtained from the perilimbal conjunctiva at the 12 o’clock position during extracapsular cataract extraction surgery. This area was chosen because it is protected from ultraviolet light by the upper lid. Specimens from pterygium and normal conjunctiva were obtained, formalin fixed, and paraffin embedded serial sections were immunostained using specific monoclonal antibodies against keratan sulfate, heparan sulfate, and dermatan sulfate. Tissue sections were incubated with mouse primary monoclonal antibodies at 4^o^C overnight. They were then extensively washed in 50 millimole tris-buffered saline, pH 7.6, before the addition of a biotinylated goat anti-mouse secondary antibody. Immunoreactivity was performed by adding 3, 3’-diaminobenzidine tetrahydrochloride (Sigma, Sydney, Australia). Following immunostaining, the tissue sections were examined meticulously, using light microscopy. A negative control of the technique was accomplished without the primary antibody. The primary antibodies used to identify the distribution of pterygium and normal conjunctival PGs/GAGs were monoclonal antibodies against heparan sulfate (clone 10E4) diluted 1:20, -keratan sulfate (clone 5D4) diluted 1:200, and dermatan sulfate proteoglycan decorin (clone 6B6) diluted 1:1000 (Seikagaku Corporation, Tokyo, Japan). Extracted data has been analysed subsequently.

## RESULTS

**Figure 1:**
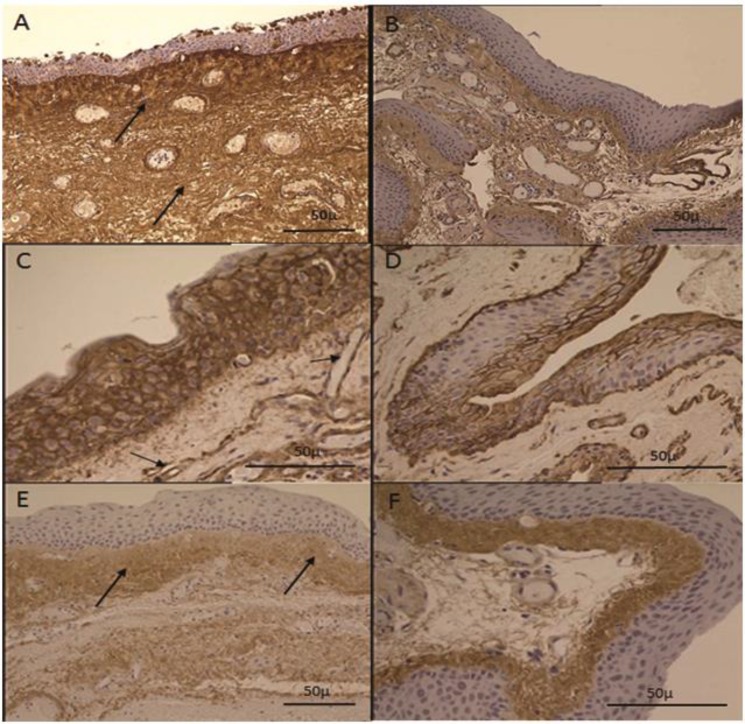
Immunolocalization and Distribution of Proteoglycans in Pterygium and Normal Conjunctiva Sections. Immunoreactivity against each Proteoglycan is denoted by arrowheads. A, B: Increased expression of keratan sulfate (arrows) in the stroma of pterygium tissue (A) compared to the normal conjunctiva (B) (×200). C, D: Overexpression of heparan sulfate (arrows) in the epithelial layer and around the capillaries of pterygium (C) compared to the normal conjunctiva (D) (×400). E, F: Overexpression of dermatan sulfate in the sub-epithelial area (arrows) of the pterygium (E) and of normal conjunctiva (F), but also in the stroma of the pterygium (×200).

Twenty-four patients (14 males and 10 females, age of 71.4 ± 6.2 years (mean ± Standard Deviation)) undergoing primary pterygium excision and 17 healthy individuals (10 males and 7 females, age 74.5 ± 8.2 of years (mean ± Standard Deviation)) undergoing extracapsular cataract surgery were included in the study. There was an increase in the expression of keratan sulfate, heparan sulfate, and dermatan sulfate in pterygium tissue compared to normal conjunctiva. More specifically, the immunohistochemical study provided evidence of increased expression of keratan sulfate in the stroma of pterygium tissue in 75% of the specimens compared to the normal conjunctiva (Fig 1A, 1B). An overexpression of heparan sulfate was observed in the epithelial layer and around the capillaries of pterygium in all samples studied (Fig 1C, 1D). Finally, immunohistochemical analysis demonstrated an increased expression of dermatan sulfate in the sub-epithelial area in the pterygium tissue and in normal conjunctiva tissue yet also throughout the stroma of the pterygium in 83.3% of the specimens (Fig 1E and 1F). The current findings were not attributed to differences in the age of patients with pterygium and normal conjunctiva as there was no statistical significant difference between the mean ages of the two groups. 

## DISCUSSION

One of the major components of the ECM are PGs that upon interactions with several growth factors and other mediators, play pivotal biological roles and determine many cellular functions, such as the cellular adhesion, proliferation, migration, and cell differentiation and apoptosis [7]. Differences between pterygium tissue and normal conjunctiva in GAGs composition has been previously identified using biochemical methods [9]. However, the expression and localization of PGs/GAGs in these tissues has never been studied previously. In the present study, using specific antibodies against certain GAG chains of PGs, the researchers identified that there were higher depositions of keratan sulfate to the tissue stroma compared to normal conjunctiva. Also, an increased expression of heparan sulfate was observed in the epithelial layer, as well as around the pterygium vessels. On the other hand, dermatan sulfate showed an increased expression and localization in the sub-epithelial layer cells of both pterygium tissue and normal conjunctiva. Dermatan sulfate, however, was also expressed in the stroma of the pterygium. All these changes were observed in a great proportion of the pterygium samples studied.

The extracellular matrix seems to play a significant role in the pathogenesis and progression of pterygium. It has been revealed that there is no difference in the cellular proliferation pattern between pterygium and conjunctival tissues, proposing that the overexpression of ECM predominates over the cellular proliferation in the pterygium pathogenesis [10]. Furthermore, Kaneto observed the presence of PGs in the tissue of pterygium [11]. Akamatsu et al. reported that chondroitin sulfate was distributed predominantly in the apical region of pterygium, where elastoid fibers were also present and suggested that degeneration of the connective tissues was responsible for connective tissue overgrowth [12]. A recent experimental study on rabbits showed that subconjunctival injection of exogenous ECM alone or in conjunction with a fibroblast cell line resulted in tissue growth with characteristics of human pterygium, a fact that highlights the role of ECM in pterygium pathogenesis [13]. It is important to note, the exogenous ECM that was injected sub-conjunctivally in the experiment contained the heparin sulfate proteoglycan. Histopathological evaluation of pterygium demonstrates a variety of findings, such as squamous metaplasia of epithelial cells and goblet cell hyperplasia and fibrovascular connective tissue overgrowth with elastotic degenerative alterations of the connective tissue, while prominent neovascularization and inflammatory cell infiltration was also observed [14, 15]. Significant changes both in the epithelium and in the underlying connective tissue and diffuse immune cell infiltrate has been shown by Golu et al. [16]. 

The current study had certain limitations. A larger number of specimens studied could give strength to the study. Although localization of the specific PGs is clearly demonstrated, calculation of color intensity of IHC stained specimens in the two groups could allow analysis of differences between the two groups in expression of the PGs/GAGs statistically and report its significance with more confidence.

## CONCLUSION

The current study clearly demonstrated differences in expression and localization of PGs/GAGs in pterygium and normal conjunctiva. Given the significant role of extracellular matrix in the pathogenesis and progression of various diseases it is therefore plausible to suggest that the overexpression and the different localization of the matrix PGs in the pterygium tissue, compared to normal conjunctiva, could contribute to tissue overgrowth, structural organization and junction of the pterygium tissue. Further studies are required in order to elucidate in detail whether certain PG populations are responsible for the pathogenicity of this disorder by evaluating the expression of mRNAs coding for the matrix and cell surface PG populations.

## References

[1] Rezvan F, Khabazkhoob M, Hooshmand E, Yekta A, Saatchi M, Hashemi H (2018). Prevalence and risk factors of pterygium: a systematic review and meta-analysis. Surv Ophthalmol.

[2] Coroneo MT (1993). Pterygium as an early indicator of ultraviolet insolation: a hypothesis. Br J Ophthalmol.

[3] Liang QF, Xu L, Jin XY, You QS, Yang XH, Cui TT (2010). Epidemiology of pterygium in aged rural population of Beijing, China. Chin Med J (Engl).

[4] Nakagami T, Murakami A, Okisaka S, Ebihara N (1999). Mast cells in pterygium: number and phenotype. Jpn J Ophthalmol.

[5] Liu L, Yang D (1993). Immunological studies on the pathogenesis of pterygium. Chin Med Sci J.

[6] Perez-Rico C, Pascual G, Sotomayor S, Asunsolo A, Cifuentes A, Garcia-Honduvilla N (2014). Elastin development-associated extracellular matrix constituents of subepithelial connective tissue in human pterygium. Invest Ophthalmol Vis Sci.

[7] Theocharis AD, Skandalis SS, Gialeli C, Karamanos NK (2016). Extracellular matrix structure. Adv Drug Deliv Rev.

[8] Karamanos NK, Piperigkou Z, Theocharis AD, Watanabe H, Franchi M, Baud S (2018). Proteoglycan Chemical Diversity Drives Multifunctional Cell Regulation and Therapeutics. Chem Rev.

[9] Kaneko M, Takaku I, Katsura N (1986). Glycosaminoglycans in pterygium tissues and normal conjunctiva. Jpn J Ophthalmol.

[10] Karukonda SR, Thompson HW, Beuerman RW, Lam DS, Wilson R, Chew SJ (1995). Cell cycle kinetics in pterygium at three latitudes. Br J Ophthalmol.

[11] Kaneko M (1987). Proteoglycans from pterygium tissues. Ophthalmic Res.

[12] Akamatu Y, Sugita Y, Mori S (1969). [Studies on the pterygium VI Histochemical studies]. Nippon Ganka Gakkai Zasshi.

[13] Zavala J, Hernandez-Camarena JC, Salvador-Galvez B, Perez-Saucedo JE, Vela-Martinez A, Valdez-Garcia JE (2018). Extracellular matrix and fibroblast injection produces pterygium-like lesion in rabbits. Biol Res.

[14] Pagoulatos D, Pharmakakis N, Lakoumentas J, Assimakopoulou M (2014). Etaypoxia-inducible factor-1alpha, von Hippel-Lindau protein, and heat shock protein expression in ophthalmic pterygium and normal conjunctiva. Mol Vis..

[15] Jaworski CJ, Aryankalayil-John M, Campos MM, Fariss RN, Rowsey J, Agarwalla N (2009). Expression analysis of human pterygium shows a predominance of conjunctival and limbal markers and genes associated with cell migration. Mol Vis.

[16] Golu T, Mogoanta L, Streba CT, Pirici DN, Malaescu D, Mateescu GO (2011). Pterygium: histological and immunohistochemical aspects. Rom J Morphol Embryol.

